# Off-hour admission and mortality in acute type A aortic dissection: A nationwide cohort of 25,608 surgically treated cases: Secondary data analysis of German hospital episode statistics from 2010 to 2023

**DOI:** 10.1016/j.xjon.2026.101729

**Published:** 2026-03-16

**Authors:** Felix Kirchhoff, Andreas Kuehnl, Christoph Knappich, Konstantin Uttinger, Matthias Trenner, Daniela Branzan

**Affiliations:** a Department for Vascular and Endovascular Surgery, School of Medicine, Technical University of Munich, Munich, Germany; b Ostbayerische Technische Hochschule Amberg-Weiden, Weiden, Germany; c Department of General, Visceral, Transplant and Thoracic Surgery, University Hospital Frankfurt am Main, Goethe University, Frankfurt, Germany; d Division of Vascular Medicine, St Josefs Hospital, Wiesbaden, Germany

**Keywords:** aortic dissection, in-hospital mortality, off-hours presentation, seasonal fluctuation, 24/7 availability, health care centralization

## Abstract

**Objectives:**

Whether admission timing influences outcomes after surgery for acute Stanford type A aortic dissection (TAAD) remains uncertain. This study examined the association between off-hour admission and in-hospital mortality in a nationwide cohort, including seasonal, weekday, and time-of-day variation.

**Methods:**

All surgically or hybrid-treated TAAD cases were identified in the German Diagnosis Related Groups database from 2010 to 2023. Multilevel logistic regression was used to evaluate factors associated with in-hospital mortality, adjusting for age, sex, comorbidity burden (Elixhauser score), annual hospital volume, and temporal variables (season, weekday, and time of admission).

**Results:**

Among 25,608 patients (median age 65 years; 62.4% male), overall in-hospital mortality was 19.0%. Mortality showed no seasonal variation after adjustment. In contrast, significant off-hour effects were observed. Weekend admissions were associated with greater mortality compared with Monday (Saturday: adjusted odds ratio [aOR], 1.20; 95% CI ,1.05-1.36; *P* = .006; Sunday: aOR, 1.25; 95% CI, 1.10-1.42; *P* < .001). Nighttime admissions (12:00 to 8:00 am) also had greater mortality than daytime admissions (aOR, 1.11; 95% CI, 1.01-1.22; *P* = .04). Greater annual hospital TAAD case volume was associated with reduced mortality (aOR, 0.93; 95% CI, 0.86-0.99; *P* = .038).

**Conclusions:**

Seasonal patterns are not related with outcomes, but weekend and nighttime admissions were independently associated with increased in-hospital mortality after TAAD repair, adding further population-level evidence to an ongoing debate. These findings support centralized 24/7 aortic emergency care, consistent team availability, and optimized regional transfer pathways to mitigate temporal disparities and improve survival.

**Figure:**
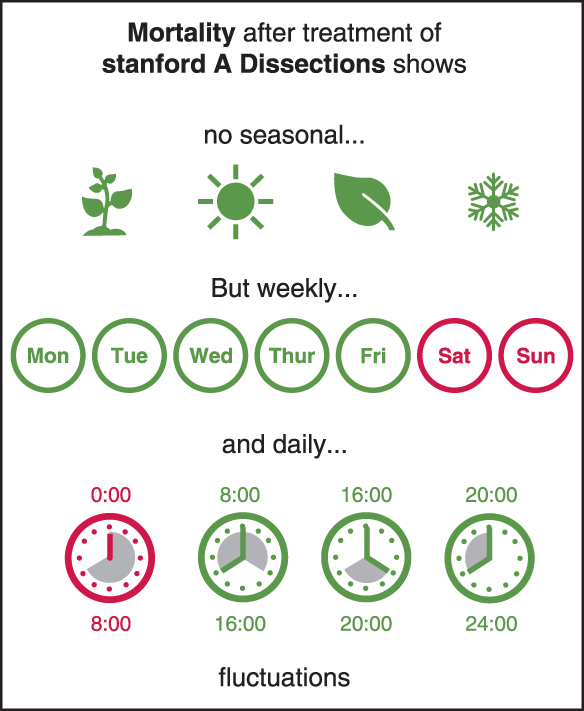
Mortality after TAAD is increased during off-hours admission but unaffected by seasonality.

**Central Message**

Mortality after acute type A aortic dissection is driven by admission timing rather than seasonality, underscoring the need for fully staffed 24/7 aortic care independent of temporal effects.

**Perspective**

Acute type A aortic dissection requires immediate, highly specialized care. Using nationwide data, this study shows that mortality is unaffected by season but significantly greater during weekends and nighttime admissions. These findings highlight the impact of organizational factors and support centralized 24/7 aortic emergency services to improve outcomes.

Acute Stanford type A aortic dissection (TAAD) is among the most lethal cardiovascular emergencies, with mortality increasing rapidly without intervention and persisting at 15% to 30% despite contemporary management.^1^^,^^2^ Timely access to highly specialized cardiothoracic surgery is therefore critical for survival.

Previous studies have reported temporal variation in the incidence of aortic syndromes, potentially related to environmental and autonomic factors.^3^, ^4^, ^5^ Similar patterns have been described for other acute cardiovascular conditions such as myocardial infarction and stroke.^6^^,^^7^

Beyond physiological triggers, organizational factors may also contribute to outcome disparities. Nationwide analyses from Germany demonstrated lower incidences and treatment rates but greater in-hospital mortality for ruptured abdominal aortic aneurysms on weekends and during night hours.^8^^,^^9^ In contrast, such “off-hour effects” were not observed in health care systems with centralized vascular services, suggesting that structural factors, including team availability and hospital volume, may mitigate temporal disparities.^10^, ^11^, ^12^

For TAAD, which requires immediate access to cardiothoracic surgery, comparable nationwide data are scarce. The present study therefore analyses seasonal, weekday, and daytime variation in the number of surgically treated TAAD cases and in-hospital mortality after treatment in Germany between 2010 and 2023, using complete nationwide Diagnosis-Related Group (DRG) data to explore both clinical and structural determinants of temporal variability.

## Methods

### Data Source and Study Design

This nationwide observational study was based on the German DRG hospital episode statistics from 2010 to 2023, provided by the Research Data Centres of the Federal and State Statistical Offices and accessed through Controlled Remote Data Processing (CRDP).^13^ The study adhered to the STROSA-2 reporting standard for secondary data analyses^14^ and the STrengthening the Reporting of OBservational studies in Epidemiology guidelines.^15^ Technical and legal aspects of CRDP and previous vascular analyses based on DRG data have been described elsewhere.^11^^,^^16^, ^17^, ^18^, ^19^ As only anonymized administrative data were analyzed, ethical approval and informed consent were not required.

### Case Identification and Cohort Definition

All inpatient cases with a main or secondary diagnosis of acute aortic dissection were identified using *International Statistical Classification of Diseases and Related Health Problems*, *Tenth Revision*, *German Modification*, codes. Surgically or hybrid-treated cases were selected based on Operation and Procedure Classification codes, as listed in Table E1. Nontreated dissections and Stanford type B cases were excluded. The primary outcome was in-hospital mortality, defined by discharge status.

### Covariates and Comorbidities

Patient characteristics included age and sex. Comorbidity burden was quantified using the Elixhauser/Van Walraven comorbidity sum score.^20^^,^^21^ Annual hospital TAAD case volume was calculated per hospital and year and log-transformed for regression analyses.

### Temporal Classifications

Season of admission was classified as spring (March-May), summer (June-August), autumn (September-November), and winter (December-February). Weekday of admission was categorized from Monday to Sunday. Time of admission was grouped into 4 periods: night (12:00-8:00 am), morning (8:00 am to 4:00 pm), afternoon (4:00 pm to 8:00 pm), and evening (8:00 pm to midnight).

### Statistical Analysis

Categorical variables are reported as absolute numbers and percentages, whereas continuous variables are reported as medians with first (Q1) and third quartiles (Q3). Because the DRG database represents a complete nationwide survey rather than a random sample, no inferential statistical testing was applied to descriptive comparisons across seasons, weekdays, or times of day (Tables 1 and 2, E2). For regression analyses, effect estimates are provided as odds ratios with 95% CIs and *P* values to indicate the strength and direction of associations.

**Table 1:** Patient characteristics of cases treated for Stanford A dissection, stratified by weekday

Weekday	Monday	Tuesday	Wednesday	Thursday	Friday	Saturday	Sunday		All patients
Treated cases (n, row-%)	4438 (17.3)	4388 (17.1)	4354 (17.0)	3942 (15.4)	3758 (14.7)	2349 (9.2)	2379 (9.3)		25,608 (100)
Characteristics									
Age, y, (quartile 1-3)	65 (55-74)	66 (55-75)	65 (55-75)	66 (56-75)	66 (55-75)	63 (54-73)	64 (54-74)		65 (55-74)
Male sex, n (%)	2789 (62.8)	2701 (61.6)	2668 (61.3)	2477 (62.8)	2315 (61.6)	1478 (62.9)	1538 (64.7)		15,966 (62.4)
Elixhauser Score, median (quartile 1-3)	12 (7-19)	12 (7-18)	12 (7-18)	12 (7-18)	11 (7-18)	11 (7-18)	11 (6-18)		12 (7-18)
Annual hospital volume, median (quartile 1-3)	30 (20-42)	30 (20-43)	30 (20-43)	30 (21-44)	30 (20-43)	29 (20-42)	30 (20-43)		30 (20-43)
Comorbidities									
Chronic ischemic heart disease	1146 (25.8)	1038 (23.7)	1120 (25.7)	1005 (25.5)	923 (24.6)	416 (17.7)	418 (17.6)		6066 (23.7)
Hypertension	3014 (67.9)	2957 (67.4)	3009 (69.1)	2649 (67.2)	2552 (67.9)	1499 (63.8)	1538 (64.6)		17,218 (67.2)
Chronic pulmonary disease	414 (9.3)	425 (9.7)	440 (10.1)	370 (9.4)	347 (9.2)	193 (8.2)	193 (8.1)		2382 (9.3)
Diabetes mellitus	419 (9.4)	404 (9.2)	437 (10.0)	386 (9.8)	341 (9.1)	182 (7.8)	184 (7.7)		2353 (9.2)
Renal disease	714 (16.1)	708 (16.1)	664 (15.3)	617 (15.7)	576 (15.3)	311 (13.2)	381 (16.0)		3971 (15.5)
Complications									
Acute myocardial infarction	173 (3.9)	168 (3.8)	170 (3.9)	148 (3.8)	148 (3.9)	107 (4.6)	103 (4.3)		1017 (4.0)
Acute kidney failure	1110 (25.0)	1082 (24.7)	1040 (23.9)	995 (25.2)	962 (25.6)	691 (29.4)	685 (28.8)		6565 (25.6)
Stroke	176 (4.0)	162 (3.7)	148 (3.4)	156 (4.0)	134 (3.6)	115 (4.9)	90 (3.8)		981 (3.8)
Acute peripheral ischemia	135 (3.0)	124 (2.8)	107 (2.5)	105 (2.7)	107 (2.9)	83 (3.5)	83 (3.5)		744 (2.9)
Mesenteric ischemia	89 (2.0)	77 (1.8)	88 (2.0)	93 (2.4)	65 (1.7)	63 (2.7)	88 (3.7)		563 (2.2)
Perioperative features									
Any valve replacement/repair	1161 (26.2)	1123 (25.6)	1108 (25.5)	974 (24.7)	865 (23.0)	530 (22.6)	527 (22.2)		6288 (24.6)
Aortic valve replacement/repair	1032 (23.3)	997 (22.7)	997 (22.9)	861 (21.8)	766 (20.4)	495 (21.1)	486 (20.4)		5634 (22.0)
Coronary bypass surgery	790 (17.8)	743 (16.9)	777 (17.9)	721 (18.3)	644 (17.1)	328 (14.0)	326 (13.7)		4329 (16.9)
Spinal catheter use	48 (1.1)	48 (1.1)	53 (1.2)	32 (0.8)	21 (0.6)	18 (0.8)	21 (0.9)		241 (0.9)
Length of hospital stay, d, median (quartile 1-3)	14 (9-22)	14 (9-22)	14 (8-21)	14 (8-21)	14 (10-21)	13 (9-21)	12 (8-22)		14 (9-21)
Mortality									
All	**823** (18.5)	769 (17.5)	810 (18.6)	744 (18.9)	707 (18.8)	489 (20.8)		515 (21.7)	4857 (19.0)
Men	486 (17.4)	461 (17.1)	463 (17.4)	435 (17.6)	444 (19.2)	287 (19.4)		316 (20.6)	2892 (18.1)
Women	337 (20.4)	308 (18.3)	347 (20.6)	309 (21.1)	263 (18.2)	202 (23.2)		199 (23.7)	1965 (20.4)

Percentages shown are in relation to column if not stated otherwise.

**Table 2:** Patient characteristics of cases treated for Stanford A, stratified by time of day

Time of day	00:00-08:00	08:00-16:00	16:00-20:00	20:00-24:00	All patients
Treated cases (n, row-%)	3851 (15.1)	13,298 (51.9)	4928 (19.2)	3531 (13.8)	25,608 (100)
Characteristics					
Male sex, n (%)	64 (54-74)	66 (56-75)	64 (54-74)	64 (54-73)	65 (55-74)
Elixhauser score, median (quartile 1-3)	2435 (63.2)	8359 (62.9)	3001 (60.9)	2171 (61.5)	15,966 (62.4)
Male sex, n (%)	12 (7-17)	12 (7-19)	11 (6-18)	11 (6-17)	12 (7-18)
Annual hospital volume, median (quartile 1-3)	31 (21-44)	30 (20-43)	30 (20-43)	30 (20-43)	30 (20-43)
Comorbidities					
Chronic ischemic heart disease	748 (19.4)	3693 (27.8)	1015 (20.6)	610 (17.3)	6066 (23.7)
Hypertension	2560 (66.5)	9148 (68.8)	3250 (66.0)	2260 (64.0)	17,218 (67.2)
Chronic pulmonary disease	341 (8.9)	1295 (9.7)	463 (9.4)	283 (8.0)	2382 (9.3)
Diabetes mellitus	315 (8.2)	1347 (10.1)	412 (8.4)	279 (7.9)	2353 (9.2)
Renal disease	577 (15.0)	2208 (16.6)	713 (14.5)	473 (13.4)	3971 (15.5)
Complications					
Acute myocardial infarction	136 (3.5)	507 (3.8)	228 (4.6)	146 (4.1)	1017 (4.0)
Acute kidney failure	1052 (27.3)	3280 (24.7)	1304 (26.5)	929 (26.3)	6565 (25.6)
Stroke	162 (4.2)	466 (3.5)	198 (4.0)	155 (4.4)	981 (3.8)
Acute peripheral ischemia	120 (3.1)	364 (2.7)	153 (3.1)	107 (3.0)	744 (2.9)
Mesenteric ischemia	99 (2.6)	262 (2.0)	116 (2.4)	86 (2.4)	563 (2.2)
Perioperative features					
Any valve replacement/repair	874 (22.7)	3727 (28.0)	989 (20.1)	698 (19.8)	6288 (24.6)
Aortic valve replacement/repair	797 (20.7)	3261 (24.5)	920 (18.7)	656 (18.6)	5634 (22.0)
Coronary bypass surgery	554 (14.4)	2582 (19.4)	753 (15.3)	440 (12.5)	4329 (16.9)
Spinal catheter use	27 (0.7)	138 (1.0)	44 (0.9)	32 (0.9)	241 (0.9)
Length of hospital stay, d, median (quartile 1-3)	13 (8-21)	14 (9-22)	14 (8-21)	14 (9-21)	14 (9-21)
Mortality					
All	777 (20.2)	2520 (19.0)	902 (18.3)	658 (18.6)	4857 (19.0)
Men	480 (19.7)	1471 (17.6)	538 (17.9)	403 (18.6)	2892 (18.1)
Women	297 (21.0)	1049 (21.2)	364 (18.9)	255 (18.8)	1965 (20.4)

Percentages shown are in relation to column if not stated otherwise.

The relationship between the season, day of the week, time of day, and hospital admission was presented descriptively, whereas the relationship with mortality was analyzed statistically using regression analysis. The null hypothesis here is that in-hospital mortality is not associated with the time of admission.

**Figure E1:**
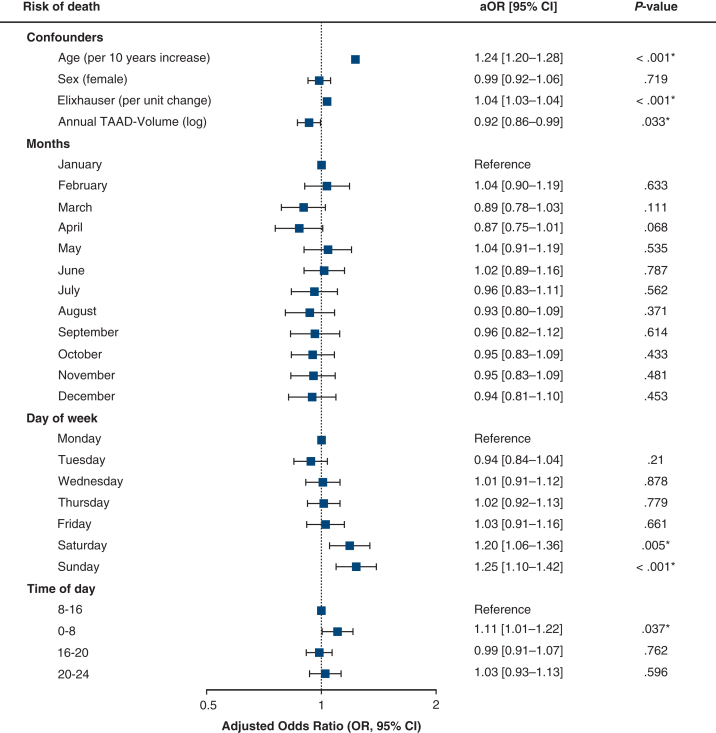
Forest plot of multilevel multivariable regression analysis of factors associated with in-hospital mortality in patients treated for TAAD. Generalized χ^2^/degrees of freedom = 0.98. ∗Statistical significance. *aOR*, Adjusted odds ratio; *TAAD*, Stanford type A aortic dissection.

Multivariable modeling was performed to identify factors independently associated with in-hospital mortality in patients treated for TAAD. A multilevel logistic regression model was implemented using the PROC GLIMMIX procedure in SAS with a logit link and binomial error distribution. On the basis of clinical relevance and prior evidence, the following covariates were included: age, sex, Elixhauser comorbidity sum score, annual hospital volume (log-transformed), and temporal variables (season, weekday of admission, time of day). As a sensitivity analysis, the multilevel logistic regression model was repeated using months (January-December) instead of seasons to evaluate whether a finer temporal granularity changed the effect estimates. The results of this monthly model are presented in Figure E1 and Tables E3 and E4.

To account for hierarchical data structure and potential clustering, random intercepts were specified for hospital and calendar year nested within hospital. This approach considers both intrahospital correlation and systematic temporal variation across observation years, providing robust standard errors and more accurate effect estimates. Model adequacy and fit were evaluated using the −2 Res Log Pseudo-Likelihood. Variance components were examined to confirm relevant between-hospital and inter-annual variability.

**Figure 1:**
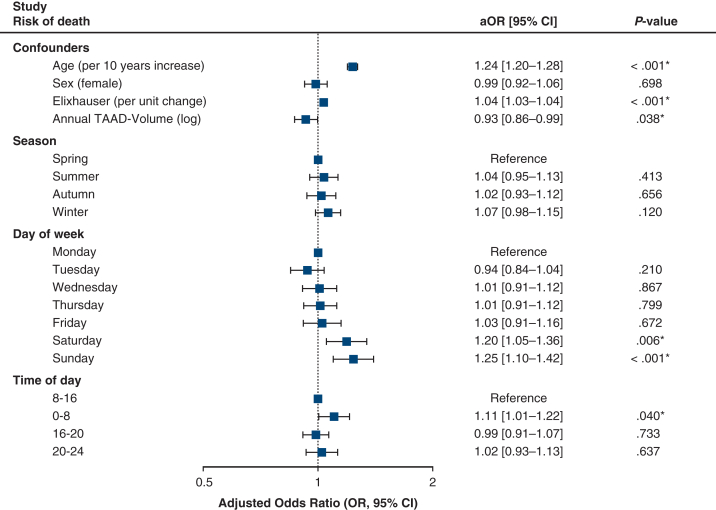
Forest plot of multilevel multivariable regressions analysis of factors associated with in-hospital mortality in patients treated for TAAD. Generalized χ^2^/degrees of freedom = 0.98. ∗Statistical significance. *TAAD*, Stanford type A aortic dissection; *aOR*, adjusted odds ratio.

Adjusted odds ratios (aORs) with 95% CI were derived to quantify associations between individual covariates and in-hospital mortality and are presented graphically as a forest plot (Figure 1). Data management and descriptive analyses were performed in SAS Studio (Release 3.82; SAS Institute Inc). Figures were generated using R (version 4.5.0; R Foundation for Statistical Computing) and Microsoft Excel. All analyses were conducted within the CRDP environment according to German standards for secondary data analysis.^14^^,^^22^

### Data-Sharing Statement

The statistical code is available from the corresponding author on reasonable request. The underlying DRG microdata are owned by the German Federal Statistical Office and cannot be shared by the authors. Access is possible for qualified researchers through the Research Data Centers according to German data protection regulations.

## Results

### Patient Characteristics

**Figure 2:**
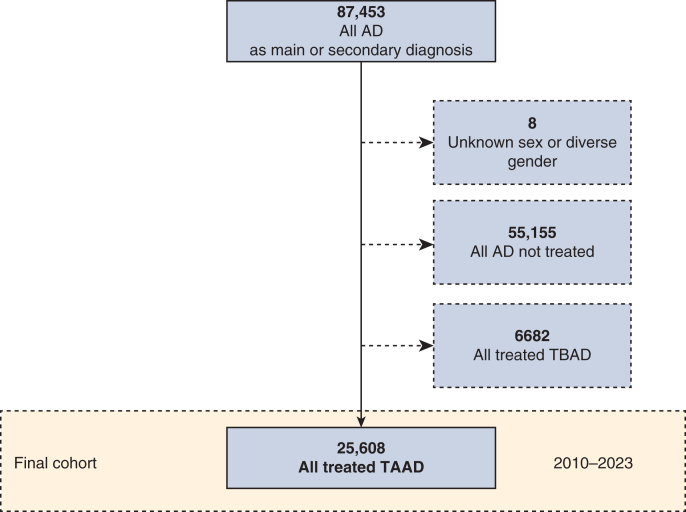
Patient flow chart. *AD*, Aortic dissection; *TAAD*, Stanford type A aortic dissection; *TBAD*, Stanford type B aortic dissection.

Between 2010 and 2023, a total of 25,608 inpatient cases undergoing surgical or hybrid-repair for TAAD were identified in the nationwide DRG statistics (Figure 2). Median age was 65 years (quartile 1, quartile 3: 55, 74 years), and 62% of patients were male. Comorbidities and procedural details are listed in Table 1. Overall, in-hospital mortality was 19.0%, with slightly greater rates among women (20.4%) than men (18.1%).

Multivariable regression analysis (Figure 1) revealed that older age (aOR, 1.24; 95% CI, 1.20-1.28, *P* < .001) and comorbidity burden (aOR per Elixhauser point 1.04; 95% CI, 1.03-1.04; *P* < .001) were independently associated with increased mortality. Greater annual hospital TAAD volume (aOR, 0.93; 95% CI, 0.86-0.99; *P* < .038) was associated with reduced mortality. Sex was not independently associated with in-hospital mortality.

### Seasonal Fluctuation

Hospital admissions were evenly distributed across seasons. Patient characteristics, procedural complexity, and complication rates did not differ in a clinically relevant extent by season (Table E2). Raw mortality rates varied slightly across seasons but showed no independent association with in-hospital mortality after risk adjustment (Figure 1). Sensitivity analyses using individual calendar months instead of seasons yielded consistent results (Figure E1 and Tables E3 and E4).

### Weekday Fluctuation

Admissions were most frequent on weekdays and lowest on weekends (Table 1). Baseline characteristics were comparable throughout the week. Crude in-hospital mortality was greatest among weekend admissions. In the adjusted analysis (Figure 1), mortality risk was significantly greater for weekend admissions compared with Monday admissions (Saturday: aOR, 1.20; 95% CI, 1.05-1.36; *P* = .006; Sunday: aOR, 1.25; 95% CI, 1.10-1.42, *P* < .001), whereas no other weekday differed significantly from Monday.

### Daytime Fluctuation

More than one half of all patients (51.9%) were admitted during regular daytime working hours (08:00 am to 4:00 pm), with the remainder presenting during afternoon, evening, or nighttime hours (Table 2). Clinical characteristics and comorbidities were consistent across time categories. Crude mortality was greatest among nighttime admissions (20.2%).

After adjustment, compared with daytime, nighttime admission (12:00 am to 8:00 am; aOR, 1.11; 95% CI, 1.01-1.22; *P* = .040) was independently associated with a greater risk of in-hospital death, whereas admissions in the afternoon and evening did not differ significantly from daytime admission (Figure 1).

## Discussion

This nationwide cohort of more than 25,000 patients surgically treated for TAAD provides the first comprehensive assessment of temporal patterns in the number of hospital cases and outcomes in Germany. No clinically relevant seasonal variation was detected, but differences by weekday and time of admission were observed. Weekend and nighttime admissions were less likely to receive concomitant valve or bypass surgery and had greater in-hospital mortality after risk adjustment. These findings suggest that temporal disparities in TAAD outcomes may primarily reflect organizational rather than biological factors within a decentralized health care system.

### Temporal Patterns and Interpretation

Our finding of no seasonal variation contrasts with previous reports describing winter peaks in acute aortic events. Atmospheric temperature and sunlight hours as well as moon phases were also discussed in other studies.^5^ In this nationwide cohort, demographic and clinical characteristics were stable across seasons, indicating that environmental or meteorological factors may play only a minor role at the population level. Similar null findings have been reported in the Netherlands,^23^ supporting the notion that climatic influences are less relevant than system-level determinants. A sensitivity analysis using individual months instead of seasons yielded comparable results, indicating that a finer temporal resolution did not change the overall findings.

In contrast, weekday and daytime variations were observed. Admissions peaked at the beginning of the week and declined on weekends, when treatment complexity and survival were lower. This mirrors earlier observations in ruptured abdominal aortic aneurysm repair^9^ and other time-critical emergencies such as myocardial infarction^7^ and stroke.^6^ Weekend admissions were associated with significantly higher mortality, in line with previous registry analyses.^4^^,^^8^

Comparable findings were reported in Japan^24^ and the United States.^25^ The reproducibility of these results across diverse health care systems supports the existence of a general “off-hours effect.”

### Mechanisms and Implications

Our findings do not resolve the controversy surrounding off-hour surgery in TAAD but add large-scale, nationwide real-world evidence that complements previous single-country and meta-analytic studies. These off-hours differences likely reflect structural limitations within a decentralized emergency care system, including reduced immediate availability of full aortic teams and restricted capacity for complex concomitant procedures during noncore hours. The markedly lower rates of valve and bypass surgery during nights and weekends in our cohort support this interpretation. Importantly, these findings align with international data demonstrating that centralized, high-volume aortic centers with guaranteed 24/7 team availability mitigate or eliminate off-hour disparities.^10^ This suggests that organizational restructuring may be an effective means of improving TAAD outcomes.

Hospital volume also remains a key determinant of outcome. Previous analyses of AAA have shown that greater annual case volume correlates with lower in-hospital mortality.^16^^,^^18^ In the current study, high-volume centers are associated with better outcomes overall, which may help attenuate—but does not necessarily eliminate—temporal disparities. Concentrating TAAD care in specialized high-volume centers, with mandatory 24/7 surgical availability, may therefore reduce temporal disparities.^26^

International experience supports this approach. The benefits of centralization of vascular services have been demonstrated in Catalonia.^27^ The consistent association between system structure and outcome highlights the potential benefit of a national aortic emergency network with coordinated referral pathways and round-the-clock team readiness.

### Limitations and Strengths

This analysis has several limitations. As in other secondary data analyses^11^^,^^19^ only patients who reached the hospital alive are included and prehospital mortality and the exact time of symptom onset remain unknown. Also, the fact that the *International Statistical Classification of Diseases and Related Health Problems* codes for “dissection” do not distinctively define Stanford A or Stanford B makes it impossible to determine the exact incidence of TAAD or Stanford type B aortic dissection because only treated cases can be analyzed separately by using the Operation and Procedure Classification codes. Another limitation is that only in-hospital mortality was recorded; follow-up was limited to the hospital stay and no 30-day or longer follow-up data were available. Coding inaccuracies are possible, but data quality is considered high due to reimbursement control by the statutory Medical Service of the Health Insurance Funds. Important dissection-specific variables such as dissection extent, hemodynamic instability, or pericardial effusion as well as surgeon-specific attributes such as surgeon volume or experience were not available in the DRG dataset, and we could not accounted for them. These factors are strong predictors of outcome in TAAD and may contribute to residual confounding. The time between admission and surgery could not be analyzed, because only the time of procedural coding is recorded but not the exact time of the procedure. Given the borderline statistical significance, the observed association with nighttime admission may be sensitive to modeling choices and residual confounding. Furthermore, the time between symptom onset and surgery could not be accounted for in the multivariable regression analysis, because this is not recorded in the underlying database. Prehospital and/or intrahospital delays likely affect TAAD outcomes and might underly temporal fluctuations (hidden confounder). Finally, residual confounding by unmeasured or undocumented clinical variables cannot be excluded. Nevertheless, the study's strengths include its nationwide coverage, large cohort size, robust hierarchical regression modelling, and consistency with established clinical evidence.

## Conclusions

In Germany, outcomes after surgery for Stanford type A aortic dissection are stable across seasons but worse for patients admitted during weekends and night hours. These temporal disparities likely reflect organizational rather than patient-related factors. Strengthening regionalization, ensuring 24/7 specialist availability, and optimizing interhospital transfer logistics may reduce these inequities and improve survival in acute aortic dissection.

## Conflict of Interest Statement

M.T. reported honoraria and travel support from Endologix, W. L. Gore & Associates, Terumo Aortic, and iVascular outside the submitted work. D.B. reported grants from Artivion, Bentley Innomed, COOK Medical, Endologix, Getinge, and Medtronic. All other authors reported no conflicts of interest.

The *Journal* policy requires editors and reviewers to disclose conflicts of interest and to decline handling or reviewing manuscripts for which they may have a conflict of interest. The editors and reviewers of this article have no conflicts of interest.

## Figures and Tables

**Table E1:** Codes for operations/procedures (OPS) and diagnosis (ICD-10-GM; source: www.bfarm.de)

Description	Source	Codes (inclusion)/reference
iAD	ICD-10	I71.00-I71.03
rAD	ICD-10	I71.04-I71.07
Treated type Stanford A	ICD-10	I71.00, I71.01, I71.03, I71.04, I71.05, I71.07
	OPS	See below
Treated type Stanford B	ICD-10	I71.00, I71.01, I71.02, I71.03, I71.04, I71.05, I71.06, I71.07
	OPS	See below
In detail type Stanford A		
I71.00	ICD-10	Dissection of the aorta, unspecified site, without mention of rupture
I71.01	ICD-10	Dissection of the thoracic aorta, without mention of rupture
I71.03	ICD-10	Dissection of the thoracoabdominal aorta, without mention of rupture
I71.04	ICD-10	Dissection of the aorta, unspecified site, ruptured
I71.05	ICD-10	Dissection of the thoracic aorta, ruptured
I71.07	ICD-10	Dissection of the thoracoabdominal aorta, ruptured
“Open repair” type Stanford A		
5-384.0x	OPS	Ascending aorta
5-384.1x	OPS	Ascending aorta with reimplantation of coronary arteries
5-384.2x	OPS	Aortic arch (until 2015)
5-384.d-f	OPS	Aortic arch (from 2016)
8-851	OPS	Cardiopulmonary bypass
5-35	OPS	Heart valve surgery
5-36	OPS	Coronary artery surgery
“Hybrid repair” type Stanford A (only in combination with 8-851, heart-lung machine)		
5-384.8	OPS	Resection of ascending aorta, aortic arch, or descending aorta with hybrid prosthesis (from 2011)
5-384.9x	OPS	Resection of ascending aorta, aortic arch, or thoracic aorta using hybrid procedure (until 2010)
5-384.bx	OPS	ascending aorta, aortic arch, or thoracic aorta using hybrid procedure (2011-2012)
5-38a.ax	OPS	ascending aorta, aortic arch, or thoracic aorta using hybrid procedure (from 2013)
In detail AD Stanford B		
I71.00	ICD-10	Dissection of the aorta, unspecified site, without mention of rupture
I71.01	ICD-10	Dissection of the thoracic aorta, without mention of rupture
I71.02	ICD-10	Dissection of the abdominal aorta, without mention of rupture
I71.03	ICD-10	Dissection of the thoracoabdominal aorta, without mention of rupture
I71.04	ICD-10	Dissection of the aorta, unspecified site, ruptured
I71.05	ICD-10	Dissection of the thoracic aorta, ruptured
I71.06	ICD-10	Dissection of the abdominal aorta, ruptured
I71.07	ICD-10	Dissection of the thoracoabdominal aorta, ruptured
“Open repair” type Stanford B		
5-384.3x	OPS	Thoracic aorta
5-384.4x	OPS	Thoracoabdominal aorta
5-384.5x	OPS	Abdominal aorta, unspecified
5-384.6x	OPS	Abdominal aorta, suprarenal
5-384.7x	OPS	Abdominal aorta, infrarenal
“Hybrid repair” type Stanford B		
5-384.ax	OPS	Thoracoabdominal aorta (bis 2010)
5-384.cx	OPS	Thoracoabdominal aorta (2011-2012)
5-38a.bx	OPS	Thoracoabdominal aorta (2013-2023)
“Endovascular repair” type Stanford B		
5-38a.70	OPS	Stent graft of the thoracic aorta: tube graft, without fenestration or side branch
5-38a.7e	OPS	Stent graft of the thoracic aorta: stent graft with coverage of one vessel branch using the chimney technique (from 2017)
5-38a.7f	OPS	Stent graft of the thoracic aorta: stent graft with coverage of two or more vessel branches using the chimney technique (from 2017)
5-38a.80	OPS	Stent graft of the thoracoabdominal aorta: tube graft, without fenestration or side branch
5-38a.8g	OPS	Stent graft of the thoracoabdominal aorta: stent graft with coverage of one vessel branch using the chimney technique (from 2017)
5-38a.8h	OPS	Stent graft of the thoracoabdominal aorta: stent graft with coverage of two or more vessel branches using the chimney technique (from 2017)
5-38a.12	OPS	Stent graft of the abdominal aorta: aortomonoiliac prosthesis without fenestration or side branch (until 2016)
5-38a.14	OPS	Stent graft of the abdominal aorta: bifurcation prosthesis, aortobiiliac, without fenestration or side branch (until 2016)
5-38a.1e	OPS	Stent graft of the abdominal aorta: tube graft, aortic, without fenestration or side branch (until 2016)
5-38a.c0	OPS	Abdominal aorta: stent graft without opening (from 2017)
5-38a.c4	OPS	Abdominal aorta: stent graft with coverage of one vessel branch using the chimney technique (from 2017)
5-38a.c5	OPS	Abdominal aorta: stent graft with coverage of two or more vessel branches using the chimney technique (from 2017)
8-849.04 + .14	OPS	Percutaneous transluminal implantation of other uncovered large-lumen stents in the aorta (04: one stent, 14: two/more stents)
8-84a.04 + .14	OPS	Percutaneous transluminal implantation of other covered large-lumen stents (04: one stent, 14: two/more stents)
5-38a.0	OPS	Aorta, not otherwise specified
Characteristics and secondary diagnosis		
Elixhauser score (ES)	Literature	van Walraven et al., Quan et al.
Cerebrovascular disease	ICD-10	I65.2
Hypertension	ES	Elixhauser Item No. 6
Chronic pulmonary disease	ES	Elixhauser Item No. 9
Diabetes	ES	Elixhauser Item No. 10 and 11
Renal disease	ES	Elixhauser Item No. 13
Secondary outcomes and complications		
ICU stay	–	Directly coded variable
Acute myocardial infarction	ICD-10	I21.x, I22.x
Acute kidney failure	ICD-10	N17.x
Peripheral arterial thrombosis and embolism	ICD-10	I74.x
Major amputation lower limb	OPS	5-864.x
Mesenteric thrombosis and embolism	ICD-10	K55.0
Cardiopulmonary reanimation	OPS	8-77x
Blood transfusion >5 units	OPS	8-800.cx without 8-800.c0

*ICD-10-GM*, *International Statistical Classification of Diseases and Related Health Problems*, *10th revision*, *German Modification*; *iAD*, intact aortic dissection; *rAD*, ruptured aortic dissection; *AD*, aortic dissection; *ICU*, intensive care unit.

**Table E2:** Patient characteristics of cases treated for Stanford A dissection, stratified by season

Season	Spring	Summer	Autumn	Winter	All patients
Treated cases (n, row-%)	6449 (25.2)	5647 (22.1)	6923 (27)	6589 (25.7)	25,608 (100)
Characteristics					
Age, y (Q1–3)	65 (55-74)	66 (55-75)	65 (55-75)	65 (55-74)	65 (55-74)
Male sex, n (%)	4049 (62.8)	3505 (62.1)	4265 (61.6)	4147 (62.9)	15,966 (62.4)
Elixhauser score, median (quartile 1-3)	12 (7-18)	12 (7-19)	12 (7-18)	12 (7-18)	12 (7-18)
Annual hospital volume, median (quartile 1-3)	30 (21-43)	31 (21-44)	30 (20-42)	30 (19-43)	30 (20-43)
Comorbidities					
Chronic ischemic heart disease	1570 (24.3)	1380 (24.4)	1647 (23.8)	1469 (22.3)	6066 (23.7)
Hypertension	4389 (68.1)	3796 (67.2)	4656 (67.3)	4377 (66.4)	17,218 (67.2)
Chronic pulmonary disease	613 (9.5)	530 (9.4)	635 (9.2)	604 (9.2)	2382 (9.3)
Diabetes mellitus	591 (9.2)	538 (9.5)	621 (9)	603 (9.2)	2353 (9.2)
Renal disease	1027 (15.9)	917 (16.2)	1046 (15.1)	981 (14.9)	3971 (15.5)
Complications					
Acute myocardial infarction	252 (3.9)	229 (4.1)	282 (4.1)	254 (3.9)	1017 (4.0)
Acute kidney failure	1668 (25.9)	1464 (25.9)	1773 (25.6)	1660 (25.2)	6565 (25.6)
Stroke	264 (4.1)	207 (3.7)	278 (4)	232 (3.5)	981 (3.8)
Acute peripheral ischemia	204 (3.2)	180 (3.2)	196 (2.8)	164 (2.5)	744 (2.9)
Mesenteric ischemia	148 (2.3)	131 (2.3)	153 (2.2)	131 (2)	563 (2.2)
Perioperative features					
Any valve replacement/repair	1632 (25.3)	1393 (24.7)	1661 (24)	1602 (24.3)	6288 (24.6)
Aortic valve replacement/repair	1465 (22.7)	1221 (21.6)	1491 (21.5)	1457 (22.1)	5634 (22.0)
Coronary bypass surgery	1070 (16.6)	1022 (18.1)	1134 (16.4)	1103 (16.7)	4329 (16.9)
Spinal catheter use	62 (1.0)	60 (1.1)	58 (0.8)	61 (0.9)	241 (0.9)
Length of hospital stay, d, median (quartile 1-3)	14 (9-22)	14 (9-22)	14 (9-22)	14 (8-21)	14 (9-21)
Mortality					
All	1193 (18.5)	1087 (19.2)	1298 (18.7)	1279 (19.4)	4857 (19.0)
Men	726 (17.9)	642 (18.3)	766 (18.0)	758 (18.3)	2892 (18.1)
Women	467 (19.5)	445 (20.8)	532 (20.0)	521 (21.3)	1965 (20.4)

Percentage is in relation to column if not stated otherwise.

**Table E3:** Patient characteristics of cases treated for Stanford A dissection, stratified by months (January-June)

Month	January	February	March	April	May	June	All patients
Treated cases (n, row-%)	2332 (9.1)	2173 (8.5)	2284 (8.9)	2098 (8.2)	2067 (8.1)	1878 (7.3)	25,608 (100)
Characteristics							
Age, y, median (quartile 1-3)	65 (55-74)	65 (55-74)	65 (55-75)	64 (54-74)	66 (55-75)	66 (56-75)	65 (55-74)
Male sex, n (%)	1499 (64.3)	1389 (63.9)	1392 (61.0)	1345 (64.1)	1312 (63.5)	1152 (61.3)	15,966 (62.4)
Elixhauser score, median (quartile 1-3)	12 (7-18)	12 (7-18)	12 (7-18)	12 (7-18)	12 (7-18)	12 (6-19)	12 (7-18)
Annual hospital volume, median (quartile 1-3)	30 (20-43)	31 (19-44)	30 (21-44)	30 (20-43)	31 (20-43)	31 (21-45)	30 (20-43)
Comorbidities							
Chronic ischemic heart disease	524 (22.5)	522 (24)	558 (24.4)	498 (23.7)	514 (24.9)	460 (24.5)	6066 (23.7)
Hypertension	1514 (64.9)	1467 (67.5)	1530 (67)	1455 (69.4)	1404 (67.9)	1274 (67.8)	17,218 (67.2)
Chronic pulmonary disease	208 (8.9)	218 (10.0)	224 (9.8)	181 (8.6)	208 (10.1)	177 (9.4)	2382 (9.3)
Diabetes mellitus	237 (10.2)	201 (9.3)	202 (8.8)	191 (9.1)	198 (9.6)	193 (10.3)	2353 (9.2)
Renal disease	366 (15.7)	346 (15.9)	381 (16.7)	325 (15.5)	321 (15.5)	306 (16.3)	3971 (15.5)
Complications							
Acute myocardial infarction	87 (3.7)	86 (4.0)	86 (3.8)	94 (4.5)	72 (3.5)	68 (3.6)	1017 (4.0)
Acute kidney failure	582 (25.0)	557 (25.6)	590 (25.8)	541 (25.8)	537 (26.0)	480 (25.6)	6565 (25.6)
Stroke	73 (3.1)	79 (3.6)	88 (3.9)	92 (4.4)	84 (4.1)	68 (3.6)	981 (3.8)
Acute peripheral ischemia	52 (2.2)	56 (2.6)	66 (2.9)	62 (3.0)	76 (3.7)	56 (3)	744 (2.9)
Mesenteric ischemia	44 (1.9)	37 (1.7)	50 (2.2)	40 (1.9)	58 (2.8)	51 (2.7)	563 (2.2)
Perioperative features							
Any valve replacement/repair	602 (25.8)	530 (24.4)	584 (25.6)	541 (25.8)	507 (24.5)	474 (25.2)	6288 (24.6)
Aortic valve replacement/repair	548 (23.5)	476 (21.9)	523 (22.9)	485 (23.1)	457 (22.1)	428 (22.8)	5634 (22.0)
Coronary bypass surgery	388 (16.6)	379 (17.4)	384 (16.8)	314 (15.0)	372 (18.0)	335 (17.8)	4329 (16.9)
Spinal catheter use	21 (0.9)	24 (1.1)	21 (0.9)	18 (0.9)	23 (1.1)	20 (1.1)	241 (0.9)
Length of hospital stay, d, median (quartile 1-3)	14 (8-21)	14 (8-21)	14 (9-21)	14 (9-22)	14 (8-21)	14 (9-21)	14 (9-21)
Mortality							
All	456 (19.6)	434 (20.0)	408 (17.9)	362 (17.3)	423 (20.5)	376 (20.0)	4857 (19.0)
Men	275 (18.3)	260 (18.7)	245 (17.6)	226 (16.8)	255 (19.4)	224 (19.4)	2892 (18.1)
Women	181 (21.7)	174 (22.2)	163 (18.3)	136 (18.1)	168 (22.3)	152 (20.9)	1965 (20.4)

Percentage is in relation to column if not stated otherwise.

**Table E4:** Patient characteristics of cases treated for Stanford A dissection, stratified by months (July-December)

Month	July	August	September	October	November	December	All patients
Treated cases (n, row-%)	1850 (7.2)	1919 (7.5)	2127 (8.3)	2320 (9.1)	2476 (9.7)	2084 (8.1)	25,608 (100)
Characteristics							
Age, y, median (quartile 1-3)	66 (56-75)	65 (55-74)	65 (55-75)	65 (55-75)	65 (55-75)	64 (55-74)	65 (55-74)
Male sex (n, %)	1149 (62.1)	1204 (62.7)	1281 (60.2)	1478 (63.7)	1506 (60.8)	1259 (60.4)	15,966 (62.4)
Elixhauser score, median (quartile 1-3)	12 (7-18)	13 (7-19)	11 (7-18)	12 (7-18)	11 (6-17)	11 (6-17)	12 (7-18)
Annual hospital volume, median (quartile 1-3)	31 (21-44)	30 (20-43)	30 (21-43)	30 (20-42)	30 (20-42)	29 (18-42)	30 (20-43)
Comorbidities							
Chronic ischemic heart disease	465 (25.1)	455 (23.7)	525 (24.7)	556 (24)	566 (22.9)	423 (20.3)	6066 (23.7)
Hypertension	1241 (67.1)	1281 (66.8)	1431 (67.3)	1552 (66.9)	1673 (67.6)	1396 (67.0)	17,218 (67.2)
Chronic pulmonary disease	165 (8.9)	188 (9.8)	192 (9.0)	209 (9.0)	234 (9.5)	178 (8.5)	2382 (9.3)
Diabetes mellitus	166 (9.0)	179 (9.3)	208 (9.8)	206 (8.9)	207 (8.4)	165 (7.9)	2353 (9.2)
Renal disease	283 (15.3)	328 (17.1)	322 (15.1)	362 (15.6)	362 (14.6)	269 (12.9)	3971 (15.5)
Complications							
Acute myocardial infarction	80 (4.3)	81 (4.2)	88 (4.1)	96 (4.1)	98 (4.0)	81 (3.9)	1017 (4.0)
Acute kidney failure	485 (26.2)	499 (26.0)	544 (25.6)	615 (26.5)	614 (24.8)	521 (25.0)	6565 (25.6)
Stroke	72 (3.9)	67 (3.5)	91 (4.3)	96 (4.1)	91 (3.7)	80 (3.8)	981 (3.8)
Acute peripheral ischemia	70 (3.8)	54 (2.8)	55 (2.6)	67 (2.9)	74 (3.0)	56 (2.7)	744 (2.9)
Mesenteric ischemia	44 (2.4)	36 (1.9)	48 (2.3)	53 (2.3)	52 (2.1)	50 (2.4)	563 (2.2)
Perioperative features							
Any valve replacement/repair	457 (24.7)	462 (24.1)	509 (23.9)	551 (23.8)	601 (24.3)	470 (22.6)	6288 (24.6)
Aortic valve replacement/repair	395 (21.4)	398 (20.7)	451 (21.2)	491 (21.2)	549 (22.2)	433 (20.8)	5634 (22.0)
Coronary bypass surgery	369 (20.0)	318 (16.6)	352 (16.6)	388 (16.7)	394 (15.9)	336 (16.1)	4329 (16.9)
Spinal catheter use	24 (1.3)	16 (0.8)	19 (0.9)	20 (0.9)	19 (0.8)	16 (0.8)	241 (0.9)
Length of hospital stay, d, median (quartile 1-3)	14 (9-21)	14 (9-22)	14 (9-22)	14 (9-21)	14 (9-21)	14 (9-21)	14 (9-21)
Mortality							
All	351 (19.0)	360 (18.8)	400 (18.8)	435 (18.8)	463 (18.7)	389 (18.7)	4857 (19.0)
Men	207 (18.0)	211 (17.5)	216 (16.9)	272 (18.4)	278 (18.5)	223 (17.7)	2892 (18.1)
Women	144 (20.5)	149 (20.8)	184 (21.7)	163 (19.4)	185 (19.1)	166 (20.1)	1965 (20.4)

Percentage is in relation to column if not stated otherwise.
